# Furosemide in pediatric intensive care: a retrospective cohort analysis

**DOI:** 10.3389/fped.2023.1306498

**Published:** 2024-01-16

**Authors:** Melany Gaetani, Christopher S. Parshuram, Donald A. Redelmeier

**Affiliations:** ^1^ Child Health Evaluative Sciences, The Research Institute Hospital for Sick Children, Toronto, ON, Canada; ^2^ Institute of Health Policy, Management and Evaluation, University of Toronto, Toronto, ON, Canada; ^3^ Interdepartmental Division of Critical Care Medicine, Faculty of Medicine, University of Toronto, Toronto, ON, Canada; ^4^ Department of Critical Care Medicine, The Hospital for Sick Children, Toronto, ON, Canada; ^5^ Department of Paediatrics, Faculty of Medicine, University of Toronto, Toronto, ON, Canada; ^6^ Center for Safety Research, Toronto, ON, Canada; ^7^ Department of Pharmacy, The Hospital for Sick Children, Toronto, ON, Canada; ^8^ Department of Medicine, Faculty of Medicine, University of Toronto, Toronto, ON, Canada; ^9^ Institute for Clinical Evaluative Sciences, Sunnybrook Research Institute, Toronto, ON, Canada; ^10^ Department of Medicine, Sunnybrook Health Sciences Centre, University of Toronto, Toronto, ON, Canada; ^11^ Sunnybrook Research Institute, Toronto, ON, Canada

**Keywords:** drug therapy, pediatrics, critical care, furosemide, EPI—epidemiology

## Abstract

**Introduction:**

Furosemide is the most commonly used medication in pediatric intensive care. Growing data indicates improved hemodynamic stability and efficacy of furosemide infusions compared to intermittent injections, thereby suggesting furosemide infusions might be considered as first line therapy in critically ill, paediatric patients. The objective of this study is to examine furosemide treatment as either continuous infusions or intermittent injections and subsequent patient outcomes.

**Methods:**

This is a retrospective cohort analysis of patients treated in a pediatric intensive care unit (ICU) over a nine year period (July 31st 2006 and July 31, 2015). Eligible patients were admitted to either the general pediatric or cardiac specific ICU for a duration of at least 6 hours and who received intravenous furosemide treatment.

**Results:**

A total of 7,478 patients were identified who received a total of 118,438 furosemide administrations for a total of 113,951 (96%) intermittent doses and 4,487 (4%) infusions running for a total of 1,588,750 hours. A total of 5,996 (80%) patients received exclusively furosemide injections and 1,482 (20%) patients received at least one furosemide infusion. A total of 193 patients died during ICU admission, amounting to 87 (6%) of the 1,482 patients who received an infusion and 106 (2%) of the 5,996 who received intermittent injections. Multivariable regression analysis showed no statistically significant decrease in adjusted mortality for patients who received furosemide injections compared to furosemide infusions (aOR 1.20, CI 0.76–1.89).

**Discussion:**

This retrospective study observed similar mortality for patients who received furosemide infusions compared to furosemide injections. More research on furosemide in the ICU could provide insights on fluid management, drug effectiveness, and pharmacologic stewardship for critically ill children.

## Introduction

Pediatric critical care requires balancing risk and benefit along with action and reaction. Critically ill patients benefit from intravenous fluids for the correction of physiological deficits and the delivery of intravenous medications: these benefits are all weighed against the risks of fluid overload. In critically ill patients, fluid overload has been associated with poorer oxygenation, more days of mechanical ventilation, increased risk of acute kidney injury, and prolonged ICU stay (1, 2). Several modalities of fluid removal can mitigate fluid overload of which the most common is intravenous furosemide (3, 4).

Furosemide exerts pharmacological effect through the sodium-potassium- chloride pump in the loop of Henle inhibiting sodium reabsorption and causing a diuresis (5). The clinical response to furosemide is determined by the delivery of furosemide to the nephron and the natriuretic response (6). Initiation, duration, dosing and delivery method for furosemide can vary significantly (7–11). In practice, intravenous delivery of furosemide occurs as intermittent injections or continuous infusions (7, 12–18). Complicating clinical factors that influence furosemide treatment decisions include hemodynamic stability, acute kidney injury, intravenous access, and physician judgement (9, 11, 19–21).

Greater understanding of practice pattern variations may help characterize the safety and benefits of intravenous furosemide (22–24). Growing data suggests improved hemodynamic efficacy of continuous furosemide infusions compared to intermittent furosemide injections (7, 20, 25). In addition, early data suggests furosemide infusions lead to a smaller cumulative dose and less overall fluid administration (7, 11). Therefore, we hypothesized that furosemide infusions might benefit critically ill, paediatric patients. The objectives of this study were to analyze intermittent intravenous injections compared to continuous intravenous infusion of furosemide for subsequent patient outcomes.

## Materials and methods

We performed a single center retrospective cohort analysis of critically ill children. The study was conducted in a quaternary center at a busy 41 bed intensive care unit that included both specific cardiac and general pediatric patients. The study was reviewed and approved by the institutional research ethics board. Eligible patients were admitted from 31 July 2006 to 30 July 2015 (9 years). We excluded patients who stayed less than 6 h or who were not treated with intravenous furosemide. The intensive care units are geographically located in the same section of the hospital and have cross over of allied health professionals.

### Furosemide treatment

Furosemide treatment was defined as an injection or an infusion based on the documented intent of the administration as previously described (4, 26). A dose was deemed an injection when identified as an “instantaneous” administration—at a single point in time. Infusions were defined as continuous furosemide administration over a documented period of time.

Furosemide infusions were further described by total separate episodes because one patient could receive one or more infusion episodes. Infusions were identified from a dose that was documented with intravenous fluid administration over several consecutive hours, suggesting the intent to provide a period of continuous administration. Individual patients treated by both injections and infusions were designated in the infusion group. An infusion episode was defined as an infusion that was spaced away from another by at least 4 h. This period of separation was chosen to incorporate a reasonable period of delayed documentation and a period beyond which any residual drug was likely flushed through the lines. Thus, the period of infusion was defined by the first and last documented administration times from the series of documented administrations of furosemide separated by less than 4 h. In contrast, the time of each individual furosemide injection was abstracted directly from the medical record.

### Patient descriptors

Additional variables were selected in advance through literature review and clinical expertise. Patient characteristics and therapies included: weight in kilograms (<5, 5–10, 10–20, 20–40, >40); length of stay in days categorized as short (<5) or long (>5); specific ICU location, (cardiac, paediatric); timing of first furosemide dose (hours); maximum Pediatric Logistic End Organ Dysfunction (PELOD) score (continuous variable); mechanical ventilation during admission (Yes, No); length of mechanical ventilation in days categorized as shorter (<3) or longer (>3); continuous renal replacement therapy (CRRT) during admission (Yes, No); duration of hemodialysis in days categorized as short (<3) or longer (>3); provision of Extracorporeal Membrane Oxygenator support (Yes, No); and duration of ExtraCorporeal Membrane Oxygenator support (ECMO) in days categorized as shorter (<5) or longer (>5), pharmacological hemodynamic support from vasopressor infusions at the time (Yes, No).

Patient medical diagnoses were also evaluated. The common congenital cardiac diseases were grouped as: hypoplastic left heart syndrome (HLHS), left ventricular outflow tract obstruction (LVOTO), transposition of the great arteries (TGA), Tetralogy of Fallot (TOF) and septal defects. Additional medical diagnoses were categorized by class or organ system to contextualize furosemide administration include: respiratory, neurological, cancer, solid organ transplant, and systemic sepsis. The available databases lacked information on line and lumen availability in children, electrolyte levels, and long term complications of furosemide administration.

### Patient outcomes

The primary study outcome was mortality comparing patients who received furosemide infusions to patients who received furosemide injections. The duration of follow up spanned the full ICU stay. Secondary outcomes included time to death between treatment groups, the frequency and type of furosemide over time, and trends in furosemide prescribing. Patient mortality (alive or dead) was directly abstracted from the dataset. Overall survival time was calculated as the difference between time of admission to ICU and time of discharge from the ICU (dead or alive). Concurrent use of furosemide and other diuretics was also abstracted including, spironolactone, chlorothiazide, and hydrochlorothiazide.

### Data sources

Data was obtained from the CIMS-Oracle database housed in the Department of Critical Care Medicine at the Hospital for Sick Children in Canada. Demographic information was directly extracted. The PELOD score was abstracted from the system using established institutional algorithms (4). Treatment with ECMO, dialysis and mechanical ventilation support was directly abstracted (along with duration). Additional vasopressor medications (epinephrine, norepinephrine, vasopressin and phenylephrine) were also identified. Data on total number of administrations, and administration free days was also collected for furosemide.

### Statistical analyses

We calculated descriptive statistics for the whole cohort and also stratified according to drug treatment. Continuous variables were reported as means along with standard deviations (SD) or medians along with interquartile ranges (q1–q3). T-tests were used to compare means between groups for normally distributed, continuous variables and the Wilcoxon rank sum test was used to compare highly skewed continuous variables. Chi- squared tests were used to compare groups for categorical variables. A multivariable logistic regression model was used to compare mortality among patients treated with intermittent injections relative to those treated with continuous infusions of furosemide. Sensitivity analysis was performed using a logistic regression model incorporating a calculated propensity score representing the likelihood of receiving a furosemide infusion. The incorporated propensity score was calculated using logistic regression to predict the likelihood of being administered a furosemide infusion. Variables included in this logistic regression were ECMO status (Y vs. N), maximum PELOD score, total furosemide injections, ICU of admission (PICU vs. CICU), and administration of inotropes. The distribution of the propensity scores between the two groups was examined and deemed roughly similar before inclusion in the final model. Statistically significant variables from the univariate analysis were included in the multivariate analysis and propensity score models (unless otherwise used in calculating the propensity score). Multicollinearity, defined as a variable inflation factor of less than 4.0, was assessed for each of the covariates and were subsequently excluded as appropriate. Statistical significance for both the univariate analysis and multivariate analysis was defined as a *p*-value less than <0.05. Given the prevailing pattern of intermittent furosemide as compared to infusion therapy (4) an enrollment ratio of 3:1 was expected. With that, a sample size calculation based on previous work (4), (alpha = 0.05, beta = 0.2, power of 80%) we aimed to study a total of 8,000 patients reflecting 6,000 in the intermittent injection group and the remaining 2,000 in the continuous infusion group.

## Results

### Overview

A total of 17,199 patients were admitted to the ICU during the study period. A total of 7,478 patient received 1 or more doses of furosemide during their entire stay (Table 1). Of the 1,954,171 total drug doses administered, furosemide infusions accounted for a total 118,438 (6%). A total of 113,951 (96%) intermittent doses of furosemide were administered and 4,487 (4%) infusions that ran for a total of 1,588,750 h (over 100 years). Furosemide was administered on a total of 31,853 patient days and concurrently administered with spironolactone in 605 (8%) patients on 2,860 (9%) patient days, with thiazide diuretics in 327 (4%) patients on 963 (3%) patient days and with both in 59 (1%) patients, on 138 (0.05%) patient days. Furosemide was administered concurrently with potassium chloride to 2,556 (34%) of patients receiving furosemide therapy on 13,349 (35%) of total furosemide patient days. Trends in furosemide use was stable over the 9-year period studied (Supplementary Appendix S1).

**Table 1:** Baseline characteristics.

	Patients receiving furosemide injections	Patients receiving furosemide infusions	*p*-value
Patients, *N*	5,996	1,482	
Age, months, median (IQR)	16 (4–80)	4 (0.5–15)	<0.001
Female, *N* (%)	2,655 (44)	653 (44)	0.903
Admission Weight, Kg, Median (IQR)	9 (5–21)	5 (4–9)	<0.001
<5 kg, *N* (%)	1,527 (25)	687 (46)	<0.001
5–10 kg, *N* (%)	1,581 (26)	443 (30)	
10–20 kg, *N* (%)	1,312 (22)	166 (11)	
20–40 kg, *N* (%)	825 (14)	100 (7)	
>40 kg, *N* (%)	751 (13)	86 (6)	
LOS, hours, median (IQR)	72 (43–141)	207 (134–310)	<0.001
PICU *N* (%)	2,798 (47)	419 (28)	<0.001
CICU *N* (%)	3,198 (53)	1,063 (72)	
Maximum PELOD score, median (IQR)	11 (10–14)	21 (12–22)	<0.001
Mechanically ventilated patients, *N* (%)	3,665 (61)	1,371 (93)	<0.001
Mechanical ventilation <days *N* (%)	2,201 (60)	274 (20)	<0.001
Mechanical ventilation, >3 days *N* (%)	1,464 (40)	1,097 (80)	
ECMO patients, *N* (%)	46 (1)	101 (7)	<0.001
ECMO <5 days, *N* (%)	30 (65)	27 (26)	<0.001
ECMO >5 days, *N* (%)	16 (35)	74 (73)	
CRRT, *N* (%)	85 (1)	83 (7)	<0.001
CRRT <3 days, *N* (%)	40 (53)	5 (6)	<0.001
CRRT >3 days, *N* (%)	45 (47)	78 (94)	
Diagnosis			
CICU			
HLHS *N* (%)	191 (3)	102 (7)	<0.001
LVOTO, *N* (%)	296 (5)	48 (3)	0.006
Septal defects, *N* (%)	831 (14)	273 (18)	<0.001
TGA, *N* (%)	186 (3)	100 (7)	<0.001
TOF, *N* (%)	350 (6)	99 (7)	0.25
PICU			
Respiratory, *N* (%)	618 (10)	77 (5)	<0.001
Sepsis, *N* (%)	251 (4)	93 (7)	0.001
Solid organ transplantation, *N* (%)	271 (5)	71 (5)	0.71
Oncologic, *N* (%)	183 (3)	37 (3)	0.3
Neurological, *N* (%)	230 (4)	10 (1)	<0.001
Pharmacotherapy			
Timing of first furosemide administration, hours, median (IQR)	37 (24–113)	63 (29–222)	<0.001
Patients with concurrent administration with inotropic/vasopressor therapy, *N* (%)	828 (14)	939 (63)	<0.001

### Furosemide therapy and associated patient characteristics

**Figure 1:**
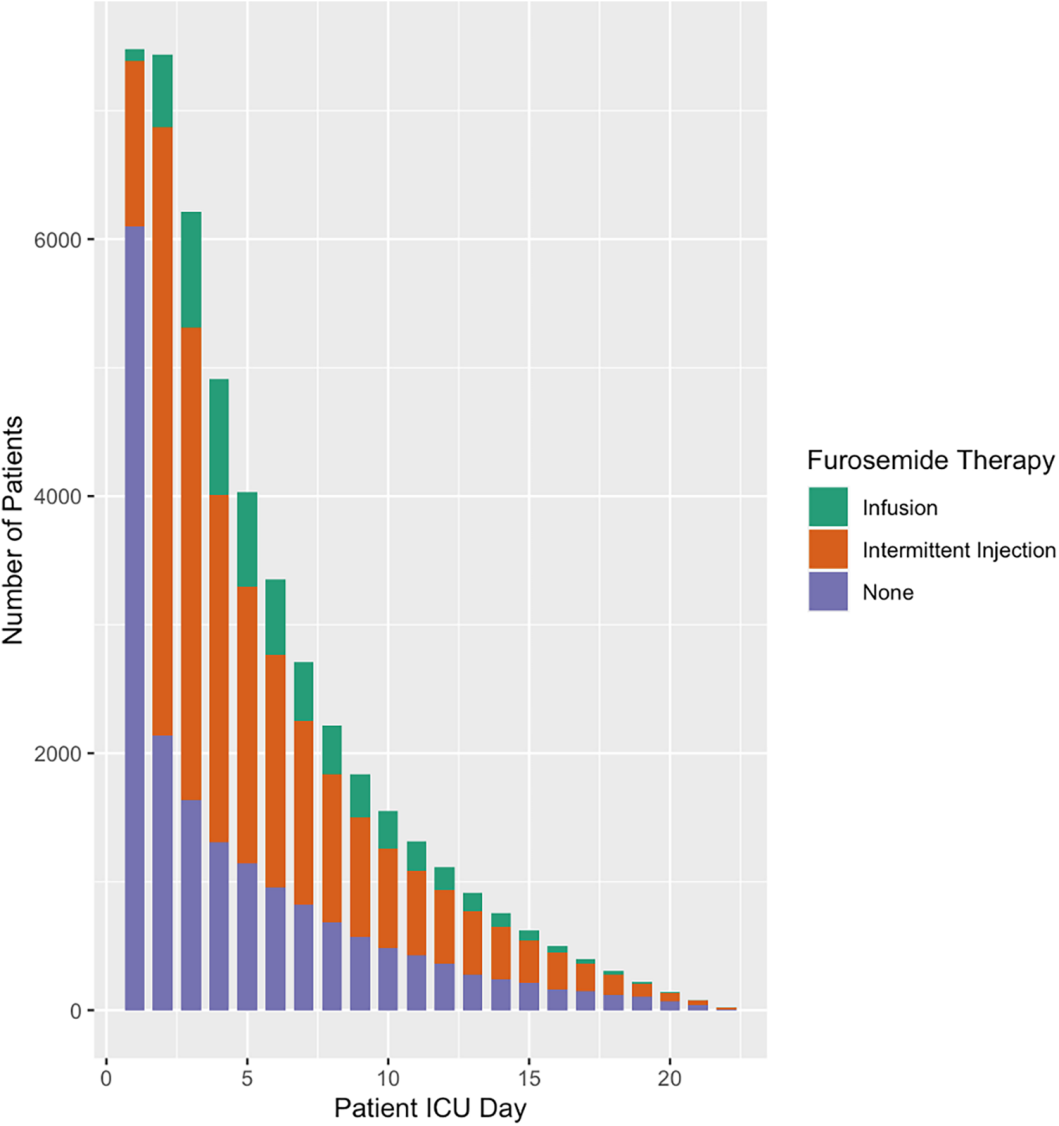
Furosemide therapy and overall use by patient ICU Day. This figure illustrates furosemide use by patient ICU Day. Most patients do not receive furosemide therapy during the first day of ICU however more than two thirds are prescribed furosemide by day 2.

**Figure 2:**
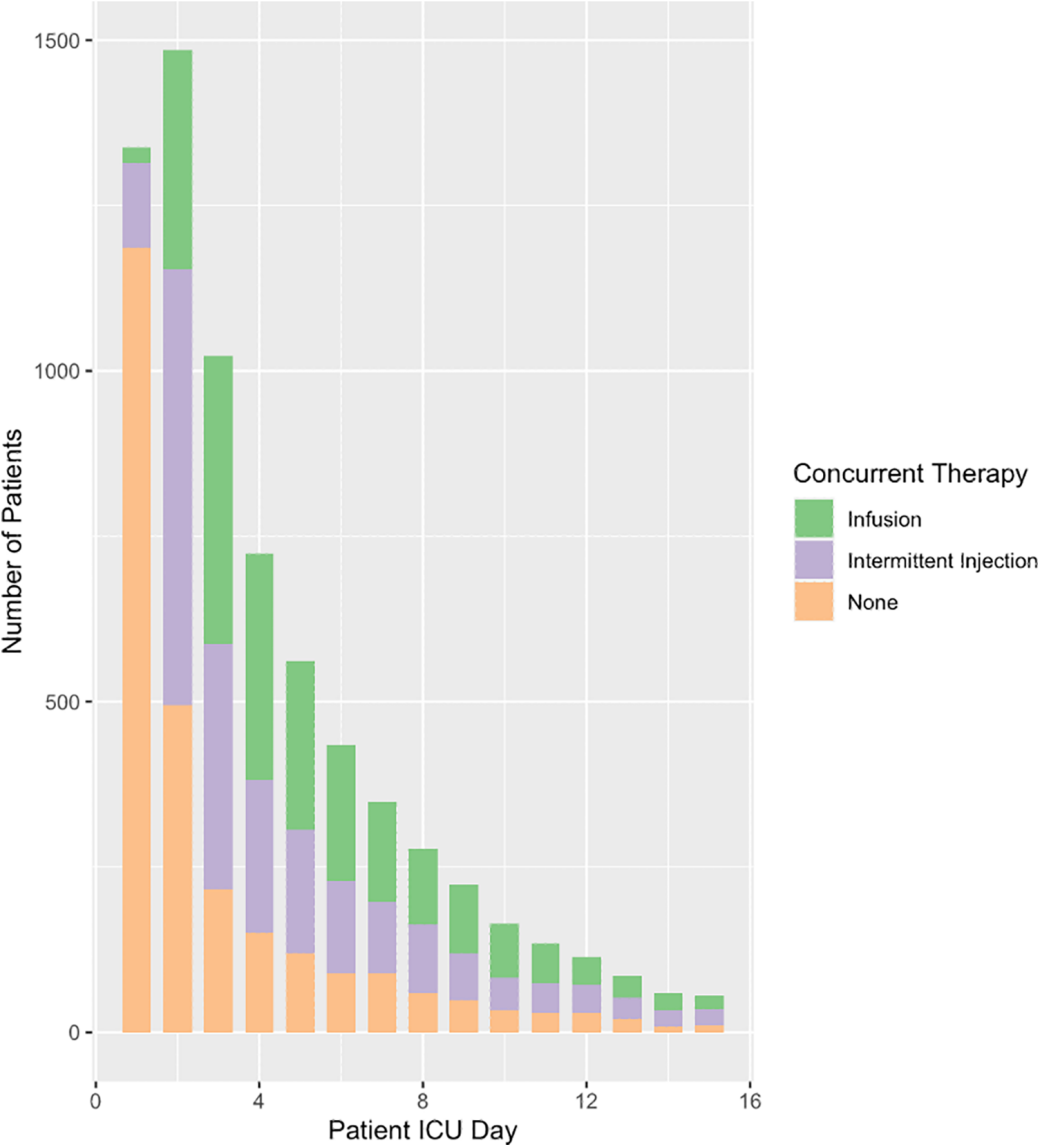
Concurrent use of furosemide and inotropic or vasopressor medications. This figure is a reflection of clinical judgement around fluid overload while concurrently managing patients with hemodynamic instability. Furosemide therapy running concurrently with hemodynamic support has important implications on initial fluid resuscitation as well as the various etiologies and subsequent physiologies associated with hemodynamic compromise. INF, furosemide infusion; INJ, furosemide injections.

Overall, a total of 5,996 (80%) patients received solely furosemide injections and 1,482 (20%) patients received at least one furosemide infusion. Almost two thirds of patients began furosemide within two days of ICU admission (Figure 1). This ratio extended to the subset of patients on hemodynamic support (Figure 2). A total of ten significant predictors related to patient characteristics, nine to therapeutic technologies, two to medications and an addition ten variables were related to primary diagnoses. A statistically significant difference was found between the two groups for all covariates except subgroups of patients with tetralogy of Fallot, cancer, and organ transplant (Table 1). The median number of furosemide injections in patients who received furosemide infusions was 10 (5–17). A total of 49 (3%) of patients who received furosemide infusions received no furosemide injections throughout ICU stay.

**Figure 3:**
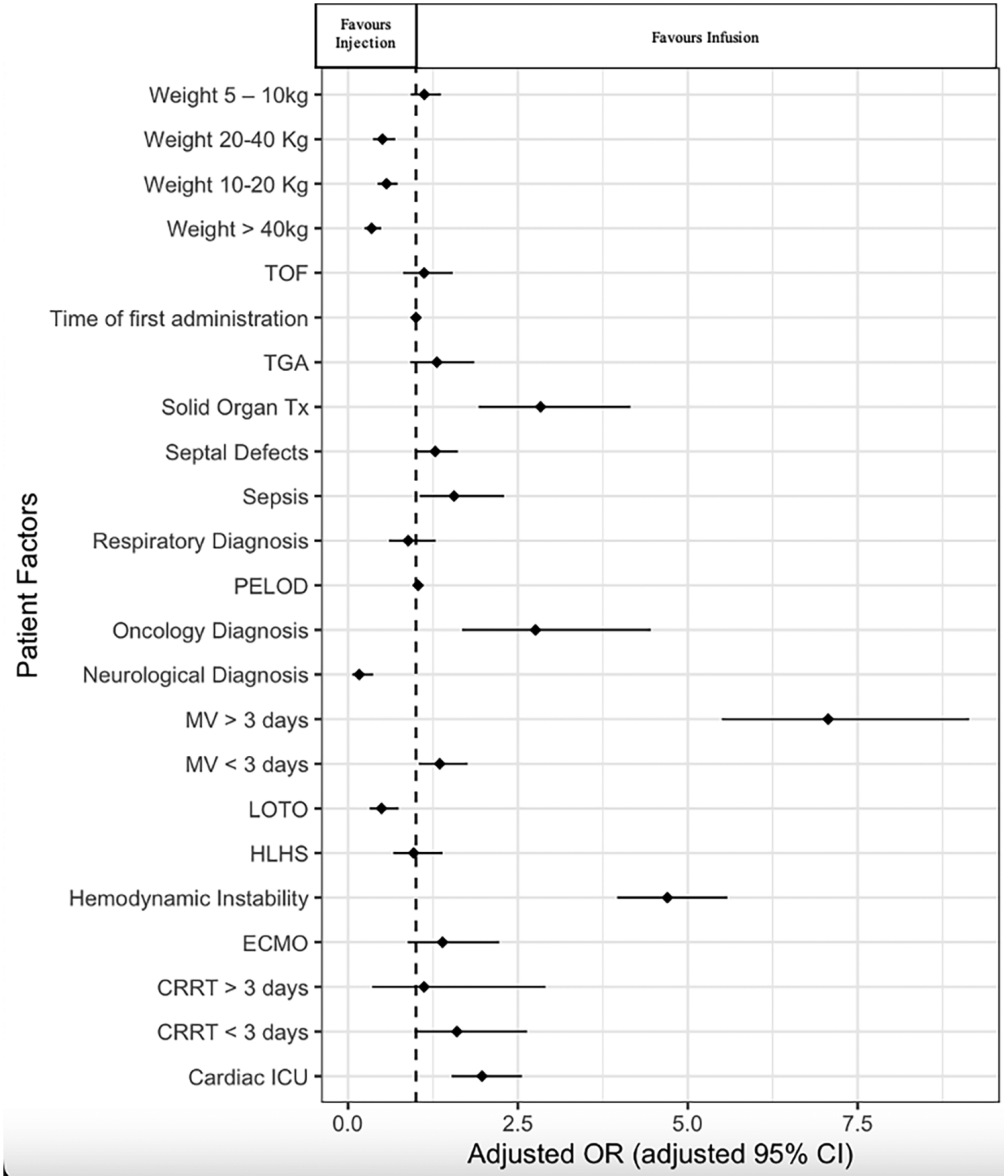
Patient characteristics and type of intravenous furosemide use. This figure demonstrates clinical and patient factors that may contribute to the use of furosemide infusions vs. injections. Patients weighing more than 10 kg are more likely to be prescribed injections. Interestingly, patients on mechanical ventilation are more likely to be prescribed infusions. TOF, tetralogy of Fallot; TGA, transportation of the great arteries; Tx, transplant; PELOD, Pediatric Logistic End Organ Dysfunction; MV, mechanical ventilation; LOTO, left ventricular outflow tract obstruction; HLHS, hypoplastic left heart syndrome; ECMO, extra corporeal membrane oxygenation; CRRT, continuous renal replacement therapy; ICU, intensive care unit.

In multivariable logistic regression all covariates were statistically significant except for the following: provision of ECMO or CRRT; patients who weight 5–10 kg; and specific diagnoses of hypoplastic left heart syndrome, transposition of the great arteries, tetralogy of Fallot or respiratory diagnoses. Patient factors and therapeutic interventions associated with decreased odds of receiving a furosemide infusion included increased weight, admission to PICU rather than CICU, a neurological diagnosis, or left ventricular outflow track obstruction (Figure 3, Supplementary Appendix S2). There was increased odds of furosemide infusion for patients with increasing PELOD scores, cancer, organ transplant, sepsis, mechanical ventilation, and hemodynamic instability (Figure 3). The final logistic regression model showed adequate fit and a c-statistic of 0.875.

### Type of furosemide therapy and patient survival

A total of 193 (3%) of patients died during ICU admission. Overall, 87 (6%) of the 1,482 patients who received an infusion died and 106 (2%) of the 5,996 that received intermittent injections died (Table 2). Univariate analysis showed a statistically significant difference between both groups for most baseline characteristics (except admission weight and age). The timing of ICU death and type of furosemide treatment on the date of death is portrayed in Supplementary Appendix S5. Most patients died within 10 days of admission with the largest number of deaths on day 3 of ICU stay.

**Table 2:** Characteristics of survival among critically ill paediatric patients.

Predictor	Patient survival		*P*- value
	Alive (*N* = 7,285)	Deceased (*N* = 193)	
Furosemide therapy			
Intermittent injections *N* (%)	5,890 (81)	106 (55)	<0.001
Infusion, *N* (%)	1,395 (19)	87 (45)	
Timing of first furosemide administration, hours, median (IQR)	40 (25–131)	75 (34–406)	<0.001
Age, median (IQR)	10 (3–66)	16 (1–143)	0.295
Weight, median (IQR)	8 (4.5–18.2)	9 (4–34)	0.460
Female, *N* (%)	3,212 (44)	96 (50)	0.137
LOS, hours, median (IQR)	94 (46–174)	161 (73–303)	<0.001
Intensive care unit			
PICU *N* (%)	3,084 (42.3)	133 (68.9)	<0.001
CICU *N* (%)	4,201 (57.7)	60 (31.1)	
PELOD score, median (IQR)	12 (10–21)	31 (22–41)	<0.001
Mechanically ventilated patients, *N* (%)	4,860 (66.7)	176 (91.2)	<0.001
ECMO patients, *N* (%)	102 (1.4)	45 (23.3)	<0.001
CRRT, *N* (%)	130 (1.8)	38 (19.7)	<0.001
Inotrope or vasopressor at furosemide initiation *N* (%)	1,639 (22)	128 (66)	<0.001
Total intermittent injections of furosemide, median (IQR)	5 (2–11)	4 (1–10)	0.032
Total infusions of furosemide median (IQR)	1.00 (1 −1)	1 (1–2)	<0.001
Diagnosis			
CICU			
HLHS	283 (3.9)	10 (5.2)	0.466
LVOTO	342 (4.7)	2 (1.0)	0.026
Septal defects	1,097 (15.1)	7 (3.6)	<0.001
TGA	285 (3.9)	1 (0.5)	0.025
TOF	449 (6.2)	0 (0.0)	0.001
PICU			
Respiratory	670 (9.2)	25 (13.0)	0.099
Sepsis	319 (4.4)	25 (13.0)	<0.001
Solid organ transplantation	333 (4.6)	9 (4.7)	1
Oncologic	204 (2.8)	16 (8.3)	<0.001
Neurological	232 (3.2)	8 (4.1)	0.589

This figure demonstrates the univariate analysis comparing patient survival and various predictor variables. A statistically significant difference was found between both groups for all predictors except admission gender, weight, age and several diagnostic categories. PICU, paediatric intensive care unit; CICU, cardiac intensive care unit; ECMO, extra corporeal membrane oxygenation; PELOD, pediatric logistic end organ dysfunction; CRRT, continuous renal replacement therapy; HLHS, hypoplastic left heart syndrome; LVOTO, left ventricular outflow tract obstruction; TGA, transposition of the great arteries; TOF, tetralogy of Fallot.

**Figure 4:**
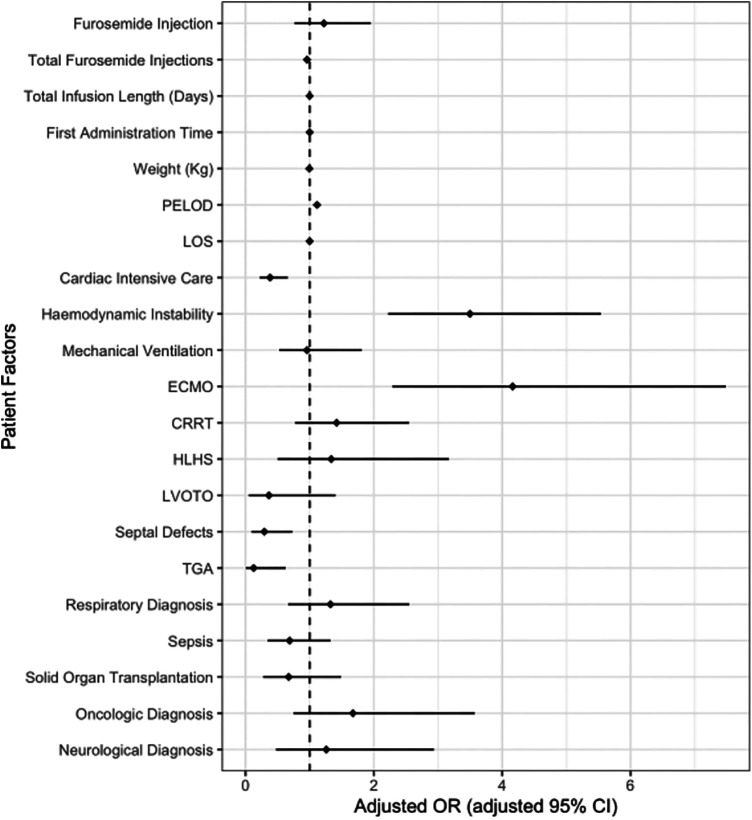
Adjusted risk of death with furosemide injections and infusions. This figure shows no difference in the odds of death between patents who receive furosemide infusions vs. injections during ICU stay after controlling for patient and therapeutic covariates. The dotted line indicates an aOR of 1 with odds rations and confidence intervals to the right of the dotted line associated with and increased odds of death. Increased odds of death were demonstrated in patients on ECMO, those who demonstrated hemodynamic instability and increasing PELOD scores. Diagnosis of tetralogy of Fallot was not included as there were no deaths in this group. ECMO, extra corporeal membrane oxygenation; PELOD, pediatric logistic end organ dysfunction; CRRT, continuous renal replacement therapy; HLHS, hypoplastic left heart syndrome; LVOTO, left ventricular outflow tract obstruction; TGA, transposition of the great arteries.

As expected, provision of ECMO, maximum PELOD score, and hemodynamic instability were statistically significant predictors of ICU mortality in the multivariate adjusted analysis (Figure 4, Supplementary Appendix S3). Total furosemide injections and admission to cardiac ICU were also statistically significant demonstrating a decreased odds of death. The final model demonstrated patients who were placed on ECMO had 5 times the odds of death compared to those without. In addition, patients who required haemodynamic support had 3 times the odds of death than their counterparts (Figure 4). There was no statistically significant difference in adjusted mortality between patients who received furosemide injections vs. furosemide infusions (Figure 4). The final model showed an adequate fit with a c-statistic of 0.936. Sensitivity analysis using a propensity score showed similar results with no statistically significant association between furosemide infusion or injection and mortality (aOR 1.39, 95% CI 0.916–2.088; *p* = 0.121, Supplementary Appendix S4).

## Discussion

We retrospectively analyzed thousands of critically ill children who received intravenous furosemide for intensive care treatment. We found that furosemide was used frequently with a total of 113,951 intermittent intravenous doses and a total of 4,487 (4%) infusions that ran for 1,588,750 h (the equivalent of nearly 200 years). In total, 193 (3%) of patients died during ICU admission. There were five main analytic implications.

First, we found no significant difference in overall mortality despite earlier evidence that suggested benefit of furosemide infusions compared to injections. Though there is a paucity of literature in the paediatric population our results are in keeping with the available adult literature and specific subsets of paediatric patients, demonstrating no difference in overall mortality in patients who receive furosemide via intermittent injection or continuous infusions (27–29). Increasing PELOD scores and hemodynamic instability were found to be associated with both an increased odds of PICU mortality and an increased odds of the provision of a furosemide infusion. Despite this overlap and controlling for these covariates, there was no difference in overall mortality in patients who receive furosemide infusions. This sheds some light on clinical practice patterns where providers could be more likely to choose continuous infusions in sicker more hemodynamically unstable patients rather than an independent risk factor for mortality alone. This work challenges the current dogma and could influence future work aiming to demonstrate the relative hemodynamic stability or instability of furosemide injections vs. infusions in the sickest patients to assess for validity and safety of the current practice.

Second, our study also demonstrates the ongoing clinical uncertainty surrounding the duration and rates of continuous furosemide infusions. There is no defined consensus for dosing of intermittent injections or continuous infusions (7–9, 11, 20, 25, 30). The rationale for infusions emphasizes improved hemodynamic stability, lower total drug exposure and maintenance of steady state excretion. Several reasons may explain why no benefit has been shown in the literature including unknown starting doses (7, 9, 11, 20), the need for ongoing titration (8, 9, 11, 20), variability in pharmacodynamics (5, 6), and poorly defined clinical outcomes (9, 11, 20, 25). As a result, the comparison of intermittent injection and continuous infusion in this study could be biased by pharmacokinetic variation in an array of clinical states as well as variation in defined clinical outcomes and end points.

Third, the similar overall outcome between furosemide therapy administration modalities in critically ill children sheds light on the safety and benefits of intermittent furosemide dosing. Critically ill children receive a median (IQR) of 19 (8–38) IV medications per patient day in the ICU and furosemide is the most commonly administered drug (4, 26). Administration of furosemide, however, has compatibility issues that can limit intravenous access (26). Liberalization of an access point by avoiding continuous infusion could aid in the provision of other necessary medications. Furthermore, furosemide infusions are one of the most commonly incompatible, concurrently administered medications (26). Eliminating incompatible concurrent drug administration through use of intermittent furosemide could ultimately improve patient safety.

Fourth, perhaps the single greatest modifiable risk factor of AKI in the pediatric ICU is the administration of nephrotoxic medications (31). Conservatively extrapolating our data on furosemide infusion and injection administrations using dosing estimates for furosemide of 1 mg/kg for injections and 0.2 mg/kg/h for infusions, the median (IQR) cumulative furosemide dose in the injection group was 4 (1–10) mg/kg vs. 28 (16–60) mg/kg in the patients who receive infusions. This could imply that some patients treated with furosemide injections are exposed to a smaller cumulative dose of furosemide (even though patients who received furosemide infusions also received injections). Future work in this area could study earlier termination of furosemide infusions compared to injections to better understand the cumulative dosing and AKI.

## Limitations

This study has many limitations. First, this was a single center analysis that justifies replication elsewhere. Second, clinical context was not extractable including nuances related to lumen and line availability or initial response to furosemide treatment. Third, detailed dosing information could provide more insight into titration techniques for furosemide infusions. Fourth, the data did not explore other consequences of furosemide administration such as clinical rationale. Fifth, have no data regarding long term outcomes of furosemide such as hearing loss. Sixth, the study cannot comment on nursing and pharmacy workload or ultimately cost effectiveness. Seventh, this is a retrospective study and associations are exploratory in nature and do not represent direct causality.

Lastly, our study does not directly examine fluid removal in response to fluid accumulation. However, the results of this study demonstrate that the majority of children in an ICU setting do not receive furosemide therapy until day 2 of admission and a significant proportion receive concurrent vasoactive medications. Fluid overload is associated with increased mortality (1, 32). Even after adjusting for illness acuity, the largest subgroup of patients demonstrated that each 1% increase in fluid overload was associated with 6% greater odds of mortality (1, 32). Given the apparent safety of concurrent furosemide treatment with inotropy in this study, earlier provision of furosemide might diminish overall fluid accumulation and improve outcomes. Further investigation through a prospective randomized control trial evaluating high vs. low dose furosemide therapy or intermittent vs. early infusion furosemide could provide a better understanding of the clinical benefits of furosemide.

This retrospective study observed similar mortality between patients who received furosemide infusions compared to furosemide injections. Further prospective work on furosemide could provide insights on fluid management, drug effectiveness, and pharmacologic stewardship for critically ill children.

## Data Availability

Data can be made available upon request in compliance with the research ethics board.
