# Supplementary material for: Furosemide in pediatric intensive care: a retrospective cohort analysis
Source: Front Pediatr. 2024 Jan 16;11:1306498. doi: 10.3389/fped.2023.1306498 (PMC10824983; doi:10.3389/fped.2023.1306498)
Supplement: Supplementary file 1 [file Table1.docx]

**APPENDICES**


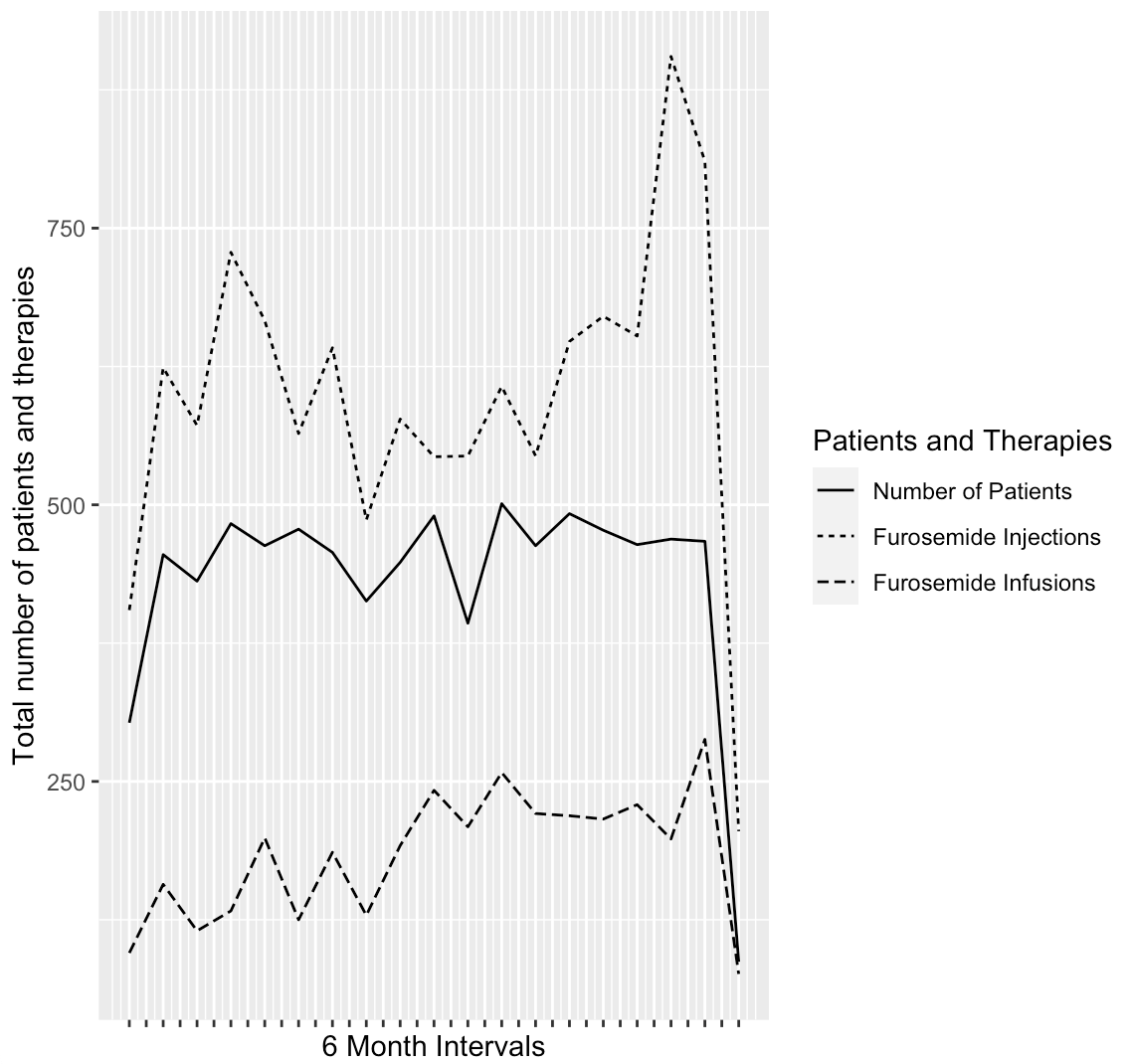
**Appendix 1: Furosemide use and trends over time**

Appendix A shows stable trends in furosemide infusions and injections over the 9-year period in accordance with patient admissions and numbers.

**Appendix 2: Patient characteristics and type of intravenous furosemide use - odds ratios and confidence intervals**

| **Variable** | **Odds Ratio** | **Lower Limit** | **Upper Limit** |
| --- | --- | --- | --- |
| Weight (5-10kg) | 1.12271995 | 0.92399839 | 1.3650048 |
| Weight (10-20kg | 0.56584179 | 0.43759012 | 0.7290154 |
| Weight (20-40kg) | 0.50697422 | 0.36707099 | 0.6946815 |
| Weight (>40kg) | 0.34730125 | 0.2440334 | 0.488709 |
| HLHS | 0.96572089 | 0.66850539 | 1.3894633 |
| LVOTO | 0.49267655 | 0.32013756 | 0.7440459 |
| Neurological Diagnosis | 0.16417494 | 0.06100139 | 0.3692687 |
| Oncologic Diagnosis | 2.75871987 | 1.6795938 | 4.4571753 |
| Respiratory Diagnosis | 0.88520779 | 0.60200553 | 1.2885901 |
| Sepsis | 1.5600401 | 1.05462571 | 2.2955903 |
| Septal Defects | 1.28340613 | 1.01869059 | 1.61553 |
| Solid Organ Transplantation | 2.83450953 | 1.92330973 | 4.1560532 |
| TGA | 1.30779022 | 0.918378 | 1.8570696 |
| Tetralogy of Fallot | 1.11962738 | 0.80961281 | 1.539619 |
| MV < 3 days | 1.34954949 | 1.04136838 | 1.7578282 |
| MV > 3 days | 7.06602689 | 5.50292745 | 9.1410647 |
| CRRT < 3 days | 1.11854874 | 0.35579484 | 2.9032261 |
| CRRT > 3 days | 1.60211815 | 0.98267689 | 2.6342262 |
| ECMO | 1.39042638 | 0.8801518 | 2.2268123 |
| PELOD | 1.03053238 | 1.02105544 | 1.0401048 |
| Cardiac Intensive Care | 1.97185428 | 1.52637714 | 2.5579458 |
| Time of First Administration | 1.00011534 | 1.00002554 | 1.0002014 |
| Haemodynamic Instability | 4.7001002 | 3.96287808 | 5.5803103 |

ECMO = extra corporeal membrane oxygenation; PELOD = Pediatric Logistic End Organ Dysfunction; CRRT = continuous renal replacement therapy; HLHS = hypoplastic left heart syndrome; LVOTO = left ventricular outflow tract obstruction; TGA = transposition of the great arteries;

**Appendix 3: Adjusted Risk of Death with Furosemide Injections and Infusions Odds Ratios and confidence intervals**

| **Variable** | **Odds Ratio** | **Lower Limit** | **Upper Limit** |
| --- | --- | --- | --- |
| Furosemide Injection | 1.222 | 0.762 | 1.953 |
| Weight (Kg) | 0.994 | 0.984 | 1.004 |
| CRRT | 1.420 | 0.774 | 2.546 |
| LOS | 1.000 | 0.998 | 1.002 |
| PELOD | 1.115 | 1.095 | 1.136 |
| Cardiac Intensive Care | 0.383 | 0.222 | 0.658 |
| First Administration Time | 1.000 | 1.000 | 1.000 |
| Haemodynamic Instability | 3.497 | 2.223 | 5.536 |
| Mechanical Ventilation | 0.958 | 0.526 | 1.811 |
| ECMO | 4.163 | 2.287 | 7.485 |
| Total Furosemide Injections | 0.959 | 0.935 | 0.981 |
| Total Infusion Length (Days) | 0.999 | 0.996 | 1.001 |
| HLHS | 1.336 | 0.501 | 3.168 |
| LVOTO | 0.365 | 0.053 | 1.401 |
| Neurological Diagnosis | 1.259 | 0.476 | 2.936 |
| Oncologic Diagnosis | 1.675 | 0.750 | 3.571 |
| Respiratory Diagnosis | 1.324 | 0.666 | 2.550 |
| Sepsis | 0.688 | 0.346 | 1.324 |
| Septal Defects | 0.294 | 0.096 | 0.730 |
| Solid Organ Transplantation | 0.673 | 0.277 | 1.489 |
| TGA | 0.128 | 0.007 | 0.627 |

ECMO = extra corporeal membrane oxygenation; PELOD = Pediatric Logistic End Organ Dysfunction; CRRT = continuous renal replacement therapy; HLHS = hypoplastic left heart syndrome; LVOTO = left ventricular outflow tract obstruction; TGA = transposition of the great arteries;

**Appendix 4: Sensitivity analysis using propensity scores for adjusted risk of death with furosemide injections and infusions - odds ratios and confidence intervals**

| **Variable** | **Odds Ratio** | **Lower Limit** | **Upper Limit** |
| --- | --- | --- | --- |
| Furosemide Injection | 1.385 | 0.916 | 2.088 |
| Weight (Kg) | 1.004 | 0.995 | 1.013 |
| CRRT | 3.891 | 2.289 | 6.516 |
| LOS | 0.997 | 0.996 | 0.999 |
| First Administration Time | 1.000 | 1.000 | 1.000 |
| Mechanical Ventilation | 1.934 | 1.107 | 3.540 |
| Total Infusion Length (Days) | 0.999 | 0.995 | 1.001 |
| HLHS | 0.755 | 0.314 | 1.597 |
| LVOTO | 0.261 | 0.042 | 0.857 |
| Neurological Diagnosis | 3.184 | 1.246 | 7.085 |
| Oncologic Diagnosis | 4.756 | 2.330 | 9.264 |
| Respiratory Diagnosis | 3.286 | 1.773 | 5.880 |
| Sepsis | 2.206 | 1.211 | 3.865 |
| Septal Defects | 0.185 | 0.064 | 0.423 |
| Solid Organ Transplantation | 1.418 | 0.615 | 2.943 |
| TGA | 0.079 | 0.004 | 0.365 |

ECMO = extra corporeal membrane oxygenation; PELOD = Pediatric Logistic End Organ Dysfunction; CRRT = continuous renal replacement therapy; HLHS = hypoplastic left heart syndrome; LVOTO = left ventricular outflow tract obstruction; TGA = transposition of the great arteries;

**
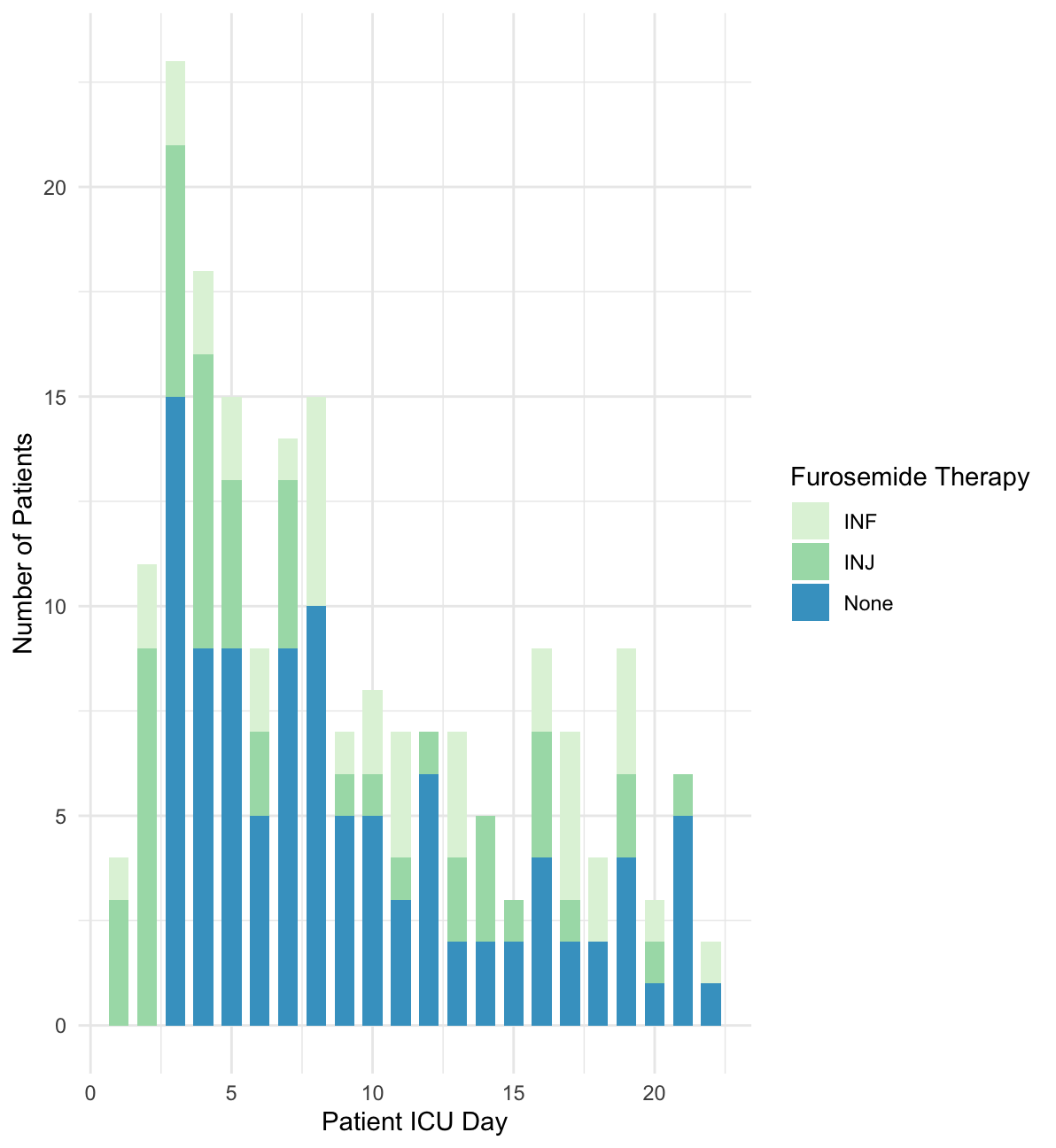
Appendix 5: Timing of death and furosemide treatment**

Figure 2 outlines the timing of death in ICU by furosemide therapy. The therapy is reflective of the type of furosemide therapy used on the date of death. A total of 193 patients (3%) died. INF = furosemide infusion; INJ = furosemide intermittent injection.
